# Robot speech: how variability matters for child–robot interactions

**DOI:** 10.3389/frobt.2025.1725423

**Published:** 2026-01-12

**Authors:** Adriana Hanulíková, Nils Frederik Tolksdorf, Sarah Kapp

**Affiliations:** Smart Cognition Lab, Institute for German as Foreign Philology, Heidelberg University, Heidelberg, Germany

**Keywords:** child-robot interactions, robot speech, robot variety, robot voice, robot-directed speech, speaking style, speech variation, variability

## Abstract

Spoken language is one of the most powerful tools for humans to learn, exchange information, and build social relationships. An inherent feature of spoken language is large within- and between-speaker variation across linguistic levels, from sound acoustics to prosodic, lexical, syntactic, and pragmatic choices that differ from written language. Despite advancements in text-to-speech and language models used in social robots, synthetic speech lacks human-like variability. This limitation is especially critical in interactions with children, whose developmental needs require adaptive speech input and ethically responsible design. In child–robot interaction research, robot speech design has received less attention than appearance or multimodal features. We argue that speech variability in robots needs closer examination, considering both how humans adapt to robot speech and how robots could adjust to human speech. We discuss three tensions: (1) feasibility, because dynamic human speech variability is technically challenging to model; (2) desirability, because variability may both enhance and hinder learning, usability, and trust; and (3) ethics, because digital human-like speech risks deception, while robot speech varieties may support transparency. We suggest approaching variability as a design tool while being transparent about the robot’s role and capabilities. The key question is which types of variation benefit children’s socio-cognitive and language learning, at which developmental stage, in which context, depending on the robot’s role and persona. Integrating insights across disciplines, we outline directions for studying how specific dimensions of variability affect comprehension, engagement, language learning, and for developing vocal interactivity that is engaging, ethically transparent, and developmentally appropriate.

## 1 Introduction

Social robots are a promising educational technology for children to support personalized and engaging learning (Peter and van Straten, 2024). However, it remains unclear how these robots should speak and sound. Spoken interaction is central to human communication and learning. In this paper, we define learning broadly as adaptive changes in children’s linguistic, cognitive, and socio-emotional skills. Because speech is both the medium and the content of much early learning, the way robots speak can directly influence how children interpret, imitate, and reason about communication itself. However, partly due to technological challenges in child–robot interaction (CRI) research, robot speech design and speech variability have received considerably less attention than other features such as appearance (Chien et al., 2025), role assignment (Rohlfing et al., 2022), or multimodal behaviors including gaze (Admoni and Scassellati, 2017) or gestures (Vogt et al., 2019; de Wit et al., 2020).

Rapid developments in artificial intelligence (AI) enable social robotics to move beyond Wizard-of-Oz (i.e., teleoperated) paradigms toward more naturalistic dialogs, using autonomously acting social robots connected to large language models (LLMs) and text-to-speech synthesis (TTS; Maure and Bruno, 2025). Synthetic speech is optimized for human-like intelligibility but usually shows less prosodic and acoustic variability than natural speech (Galdino et al., 2024). Social robots equipped with LLMs enable children to interact and learn through synthesized speech in the physical world instead of a virtual space. This disconnect between natural and synthetic speech generated by TTS technologies and LLMs raises a key design problem. Which kinds of speech variability support children’s learning across developmental stages and varying language proficiencies, in which contexts, and how can technically standardized voices be balanced with pedagogical and ethical needs for natural variation?

This perspective paper advocates a child-centered and bidirectional approach to robot speech design. Robot speech should exhibit variability but not mimic human speech in all its facets. It should be designed to adapt to children’s communicative requirements, contexts, and preferences. In what follows, we discuss insights from speech perception research, developmental psychology, and CRI studies to propose directions for studying how vocal design and persona, that is the robot’s designed social and communicative character, can support learning, trust, and developmental appropriateness in CRI.

## 2 Human speech variability: functions and implications for children’s learning

To understand how robot speech could or should vary, it is necessary to first consider the functions of the remarkable variability in human speech (e.g., Hawkins, 2003). Across and within speakers we encounter varying speaking rates, different levels of formality, reductions, disfluencies, repairs, diverse accents and dialects, and rich prosodic patterns. Speech also conveys social and indexical meaning such as speaker identity, age, emotions, socioeconomic and cultural background (e.g., Eckert, 2019). Speech variability is particularly relevant for children’s ability to robustly discriminate speech sounds, segment words, acquire vocabulary and grammar, master pronunciation (e.g., Cristia, 2013; Rowe, 2008), as well as to learn pragmatic skills such as turn-taking and conversational repair.

For developing learners, this variability strengthens linguistic representations and supports generalization. Studies show that input variability influences the acquisition of phonology (Lively et al., 1993; Sadakata and McQueen, 2013; Hanulíková, 2023), vocabulary (Barcroft and Sommers, 2005; Levy and Hanulíková, 2023), and morphosyntax (Eidsvåg et al., 2015; Gómez, 2002). Exposure to diverse language varieties provides redundant acoustic information that supports robust word recognition and facilitates generalization to new contexts (e.g., Potter and Saffran, 2017; Hanulíková and Ekström, 2017; Levy et al., 2019; Hanulíková and Levy, 2025).

To ground the discussion in concrete empirical examples, Table 1 maps speech variability dimensions to learning functions, risks, and design implications for CRI. The rows address different linguistic levels (phonetic/phonological, lexical/(morpho)syntactic, pragmatic, and discourse) where variability has multiple forms or functions and specifies its developmental relevance. For example, phonetic variability (row 1) supports attention and word segmentation (e.g., Cristia, 2013; Potter and Saffran, 2017), lexical diversity (row 2) enhances semantic networks (e.g., Hadley et al., 2019), while pragmatic features (row 3) like disfluencies can contribute to engagement but risk over-attribution of competence (e.g., Wigdor et al., 2016).

**TABLE 1:** Summary of possible key design dimensions for speech variability in child–robot interaction, integrating functional and formal perspectives. The relevance and suitability of these dimensions will vary with children’s age, learning stage, and interactional context.

Linguistic dimension	Examples of variability	Learning-relevant functions for children	Risks/challenges	CRI speech design implications
Phonetic/phonological	Pitch, rhythm, stress, timing, acoustics of sounds, pauses, disfluencies, hesitation markers, voice, accents, dialects	Increases attention and motivation, supports word segmentation, scaffolds phonological representations, adaptation and generalization, improves turn-taking, supports social inclusivity through exposure to diverse speech patterns	Overstimulation, confusion if inconsistent, timing errors disrupt interaction, limited TTS control	Adaptive modulation by task and age, include modeled disfluencies, adjustable latency
Lexical/(morpho)syntactic	Lexical diversity, gender and case marking, contextual reduction, sentence and syntactic complexity	Enhances generalization, strengthens semantic networks, supports generalization to novel forms	May hinder comprehension for low-proficiency learners, uneven benefits across developmental stages	Scaffold input from simplified to varied and more complex linguistic forms
Pragmatic/discourse	Style, register, expressiveness, pauses, disfluencies, hesitation markers, turn-taking, cultural variation (e.g., repair strategies, politeness conventions, backchannel rate)	Supports engagement, trust, and role understanding, improves conversational naturalness, supports conversational grounding across speakers and contexts	Over-attribution of competence, risk of deception if too human-like, risk of over- or under-representing certain communicative styles, leading to social or cultural bias. Recognition errors	Maintain transparency about artificial agency, use of mechanomorphic but expressive voices, curate balanced, diverse speech databases, context-sensitive pragmatics

Speech variability serves not only learning but also social-cognitive functions. It conveys socio-indexical information that signals intentions, emotional states, and group affiliations (e.g., Kinzler, 2021). Children use these cues to guide social decisions, such as preferring speakers of their own language variety or local accent over speakers of other varieties (Kinzler, 2021), a pattern observed across developmental stages and bilingual contexts (Byers-Heinlein et al., 2017; Hanulíková, 2024).

These developmental characteristics distinguish children from adult robot users: ongoing language acquisition makes input quality critical, developing social cognition makes children particularly responsive to vocal cues about trustworthiness and competence, and their limited understanding of artificial agents makes transparency essential (Sharkey and Sharkey, 2021). Seen through this lens, the dimensions summarized in Table 1 reflect not fixed design recommendations but developmentally contingent resources.

Importantly, the effectiveness of variability depends on its type, source, timing, and relevance to the learning task (Raviv et al., 2022). Rost and McMurray (2010) found that variability along linguistically irrelevant dimension (e.g., speaker voice characteristics) helps learners identify the dimensions of input to attend to, as opposed to those they can ignore. Moreover, the type of “useful” variability may depend on the stage of learning, so that in the very early stages, variability along linguistically irrelevant dimensions is most beneficial, while later, variability along relevant dimensions become more useful (Lev-Ari, 2018). A well-known example is child-directed speech (CDS), where adults and older children systematically adjust prosody, speaking rate, lexical and syntactic complexity when speaking to toddlers (Rowe, 2008; Cristia, 2013; Kempe et al., 2024). CDS demonstrates that adaptive variability serves as an implicit scaffold for learning, supporting attention (Soderstrom, 2007), emotion regulation (Singh et al., 2002) and language development (Rowe, 2008). In designing robot speech, a similar principle could guide synthetic voices toward functional rather than fully human-like variability.

Given these learning benefits, it is unclear whether synthetic speech in social robots can become its own communicative variety, a *robot variety*, that incorporates functional variability, developmental tuning, while remaining transparent about its artificial nature. Current implementations of robot speech are relatively uniform and barely dynamically adjust to a child’s abilities (Romeo et al., 2025; Kory-Westlund and Breazeal, 2019; Rohlfing et al., 2022). Thus, the question is whether the benefits of natural variability reported in speech perception research can inform the design of robot speech. Moreover, the design of robot speech must consider not only acoustic and prosodic variability but also the alignment of verbal and nonverbal behaviors, particularly as these factors interact with children’s developmental stages and interactional contexts (Wróbel et al., 2023).

## 3 Robot speech in CRI

### 3.1 Current technological implementations and challenges

Recent progress in neural TTS synthesis has made it possible to generate speech and voices that sound natural to humans (Le Maguer and Cowan, 2021). However, these systems have several limitations. Most are trained on monologic data such as audiobooks, which poorly match conversational speech (Moore, 2019). As a result, they struggle to reproduce conversational dynamics, disfluencies, and prosodic variability characteristic of spontaneous interactions (Moore and Nicolao, 2017; Moore, 2020). Temporal features such as response latency, pause placement, and overlap timing are not merely technical constraints but meaningful interactional signals that children use to infer understanding and agency. Moreover, existing models tend to suppress natural variation in speaking style, accent, dialect, register, and persona (Le Maguer and Cowan, 2021; Moore, 2019). Field studies confirm that accent mismatches can disrupt child–robot interaction and hinder learning outcomes (Singh et al., 2023). Although work on incremental language generation and speech synthesis creates more natural turn-taking through pauses, repetitions and repair (Buschmeier and Kopp, 2018), most current systems still lack the dynamic variability that characterizes human communication (Ekstedt and Skantze, 2022).

A further challenge concerns child-specific interaction. Automatic speech recognition systems still struggle with children’s variable or developmentally atypical speech patterns (Kennedy et al., 2017; Janssens et al., 2025), and generative models lack sufficient child-directed training data. Developing more ecologically valid models requires high-quality, annotated interactional datasets that capture real-world dialogic dynamics. While recent advances in child speech recognition are promising (Janssens et al., 2025), substantial data scarcity representing diverse children persists.

These technical constraints not only affect recognition accuracy but also influence how spoken interactions unfold in practical settings. Following Moore (2019), the mismatch between human-like speech and limited linguistic and interactional abilities of robots creates what he calls the “habitability gap”. If a robot sounds too human-like, children and adults may overestimate its abilities and understanding, leading to disappointment when expectations are not met. This gap is particularly problematic in CRI, where unnatural dialog patterns reduce ecological validity compared to children’s everyday language experiences, which involve highly dynamic, multimodal, and interactionally contingent input (Goldenberg et al., 2022). Studies addressing these limitations show that strategically implemented disfluencies and conversational fillers can improve turn-taking dynamics and social engagement in CRI (Ohshima et al., 2015; Wigdor et al., 2016). Nevertheless, most current systems lack the ability to adapt speech rate dynamically to children to facilitate comprehension, and they lack “priors”, that is, built-in understanding of how language works in human communicative contexts (Moore, 2005).

Because of these challenges, some researchers suggest the use of synthetic speech as a distinct, purpose-built variety. Le Maguer and Cowan (2021) argue for “natural non-human-like speech synthesis”, i.e., voices that are intelligible and expressive but transparently artificial, while Moore (2017) proposes “mechanomorphic” designs emphasizing congruency between voice and robot’s non-human identity. Marge et al. (2022) highlight this approach to align appearance, capabilities, and voice. Moreover, their work identifies the need for interaction styles to be deliberately engineered and tuned for specific scenarios, emphasizing the role of prosody in turn-taking, grounding, or conveying stance. Moore (2017) frames this as developing a “science of vocal interactivity”, referring to a systematic investigation of how vocal design in embodied agents affects learning, trust, and social dynamics. These perspectives mainly address voice design, and the focus lies primarily on general robot users. Our proposal extends this discussion in two ways: First, we apply these ideas to developmentally appropriate robot speech varieties for children in interactional and educational settings. Second, we distinguish between voice (timbre, pitch) and speech characteristics (speech rate, prosodic and articulatory variability). For interactions with children, the challenge extends beyond naturalness. Developmentally appropriate robot speech must balance familiarity with transparency: voices that sound too human-like risk eliciting misplaced trust or over-attribution of understanding, whereas overly mechanical voices can reduce engagement and warmth. An alternative is a synthetic yet expressive voice that may best support learning, engagement and trust while signaling artificiality. Such “mechanomorphic” or hybrid voices could adapt prosodic range, rhythm, and affect to the communicative context without simulating a specific human identity. Addressing this challenge calls for a systematic investigation of both dimensions (voice and speech variability) and their interaction.

### 3.2 Robot speech effects on children

Research examining how robot speech characteristics affect children shows that expressive speech enhances engagement and learning. Preschoolers interacting with robots using expressive speech showed improved word production, better narrative recall, and greater engagement in storytelling tasks compared to those interacting in monotone speech (Kory-Westlund et al., 2017; Conti et al., 2019). Similar effects have also been observed in young adults, with L2 learners performing better in a linguistic task when a robot delivers instructions in a charismatic speaking style (Fischer et al., 2021). In addition, adaptive features such as entrainment, where the robot adjusts pitch, rate, and volume to match a child’s speaking style, can support rapport and positive emotions during interaction (Kory-Westlund and Breazeal, 2019). However, when robots sound more human-like, children show greater compliance with their requests (Romeo et al., 2025), raising ethical questions about transparency and possible manipulation.

Children sometimes prefer robots that make systematic errors, which create opportunities for correction and scaffolding (Förster et al., 2023). After interacting with a robot having pronunciation difficulties, preschoolers engaged in metatalk about the robot’s voice and limitations, demonstrating emerging critical technological thinking (Tolksdorf et al., 2024). Such behaviors, including repair, clarification requests, and delayed responses constitute a critical dimension of speech variability that shape how children interpret competence, intentionality, and transparency in interaction. This suggests that strategic imperfection can support both learning and increase awareness of technology.

Studies on robot-assisted language learning show mixed outcomes. While some studies report improvements in pronunciation, vocabulary, and communicative ability (Lee et al., 2011; Wang et al., 2013), others find effects limited to listening improvements (In and Han, 2015). These inconsistencies likely reflect both varied assessment methods and the limited natural variation in TTS systems, which constrains intonation and reduces alignment opportunities for learners (Rosenthal-von der Pütten et al., 2016).

Thus, expressive, contingent, and socially responsive robot speech can promote engagement and learning, though effects remain context-dependent and methodologically fragmented.

### 3.3 Bidirectional adaptation and individual differences

Speech adaptation is central to CRI and learning because it both reflects interlocutors’ expectations about the robot’s communicative behavior and directly affects the structure of the input available for learning. Thus, child-robot interaction needs to be considered as a bidirectional process. Both adults and children modify their speech when addressing robots, using robot-directed speech (RDS). RDS is characterized by features such as slower rate, repetitions, simple sentence structure, and increased pitch variation (e.g., Breazeal, 2002; Cohn et al., 2021; Cohn et al., 2024), features similar to CDS. These modifications reflect assumptions about the robot’s cognitive and linguistic capabilities (Fischer et al., 2011) and its perceived competence rather than anthropomorphism: less capable robots elicit stronger speech adjustments (Cohn et al., 2024; Kalashnikova et al., 2023). Adults show prosodic and phonetic alignment with synthetic voices (Zellou et al., 2021; Offrede et al., 2023) and even converge on emotional expressiveness (Cohn et al., 2021).

Children also show phonetic accommodation to robot speech, adjusting fundamental frequency, vowel duration, and vowel quality, with considerable individual variation predicted by personality traits and perception of the robot’s persona (Hong and Chen, 2024). Cohn et al. (2024) showed that children’s vocal modifications are more extreme than adults’, demonstrating that age is a critical factor in RDS. Such adaptation occurs at implicit, cortical levels. Sivridag and Mani (2025) found that 5-year-olds’ brains tracked both synthesized robot speech and natural adult speech, though with longer processing delays for robot speech, indicating cortical entrainment during child-robot interaction.

Interestingly, children’s RDS may reflect interpersonal dynamics. Velner et al. (2024) found that children’s vocal characteristics (pitch variation, intensity) correlated with their trust in the robot, though effects were small. Sanoubari et al. (2024) demonstrated that prosody can disambiguate spoken input during human-robot interaction, with participants using distinct prosodic patterns to convey different intentions with identical words (e.g., “nice” meaning “keep going” vs. “stop”). This suggests that the design of robot speech and the capability of systems to reliably perceive children’s speech patterns must account for how children adapt to robots, not just how robots adapt to children.

Just as caregivers adjust their speech to individual children, robots should ideally do the same. Research on CRI has only begun to explore such adaptive patterns. While children’s vocabulary size, phonological memory, and selective attention moderate robot-assisted learning outcomes (Van den Berghe et al., 2021; Rudenko et al., 2024), few studies systematically tailor robot speech to these differences. In contrast to some work with adults (Crumpton and Bethel, 2016; Skantze et al., 2019), existing CRI learning studies (e.g., Vogt et al., 2019) tend to prioritize other communicative modalities, such as the effect of gestures. Consequently, designs that systematically adapt robot speech parameters such as speaking rate, prosody, or disfluency to individual developmental needs remain underexplored and technically limited, despite children’s vocal accommodation providing a potential signal for adaptive robot behavior.

## 4 Should robots embrace speech variability?

The preceding sections highlight speech variability as both an opportunity and a challenge for CRI, giving rise to three interrelated tensions. The first concerns feasibility, because modeling dynamic human speech variability remains technically challenging. While LLM-driven speech synthesis enables prosodic and persona-level variation, fine-grained phonetic timing or natural disfluencies still pose an issue.

The second tension concerns desirability, because speech variability can shape engagement, trust and learning in positive and negative ways. Variability strengthens linguistic representations when appropriately structured (Rost and McMurray, 2010), and expressive robot voices enhance engagement and language learning (Kory-Westlund et al., 2017; Conti et al., 2019). However, excessive or poorly timed variability risks confusion. The optimal degree of variability requires balance: too little sounds mechanical, too much undermines intelligibility and attention. Finally, for certain language-learning contexts, fine-grained phonetic variability may be equally important as prosodic expressiveness or persona cues, particularly because these dimensions interact in shaping how children perceive and adapt to robot speech, though systematic comparisons are lacking.

The third tension concerns ethics. When synthetic speech becomes indistinguishable from natural human speech, it risks obscuring a robot’s actual capabilities, leading to deception and over-attribution of competencies (Sharkey and Sharkey, 2021). Transparently synthetic yet engaging speech makes the robot’s capabilities and artificial nature clear while supporting meaningful interaction (Moore, 2017). Additional risks include increased emotional attachment and unintended reinforcement of stereotypes.

These tensions demonstrate that speech variability is not merely a technical or aesthetic concern but one with direct developmental, pedagogical, and ethical implications. We therefore define robot speech varieties as systematic, adaptive synthetic voices that remain transparently artificial but incorporate functional variation to support specific learning and communicative goals. Thus, we do not refer to a stable linguistic system similar to a human dialect, but to a principled design abstraction specifying task- and age-sensitive speech parameters. Based on proposals for “natural non-human-like speech” (Le Maguer and Cowan, 2021) and “mechanomorphic” voices (Moore, 2017), an ethical design of robot speech emphasizes transparency about the robot’s technological nature while implementing variation that support children’s development and engagement and is aligned with the robot’s role. The key question shifts from whether robots should embrace human variability to which types (phonetic and phonological features, persona characteristics, temporal dynamics and their interactions) benefit which learning goals and at what stage during development. Importantly, not all variability can be implemented at the acoustic signal level, some forms are better realized through interaction management, turn-taking strategies, or repair policies, given current system limitations. Future studies should experimentally test these parameters across developmental groups.

## 5 Discussion and looking ahead

By integrating insights across disciplines, we suggest that targeted implementation and accommodation of speech variability can be a valuable strategy in CRI, provided it is informed by technical feasibility, cognitive and contextual demands, and ethical transparency. While prior work has addressed general human–robot interactions (Marge et al., 2022; Huang and Moore, 2025), our perspective focuses on children’s specific needs, including developmental appropriateness, child-directed variability, and age-sensitive design. Addressing these questions requires systematic research across multiple linguistic dimensions which exhibit variability (see Table 1). Across the dimensions discussed, we argue that speech variability can support learning, engagement, and socio-communicative development, but that its effectiveness depends on children’s developmental stage, interactional context, and the robot’s role, persona, and capabilities. Overgeneralizing from human speech variability risks misrepresenting robot competence; therefore, variation should remain functional, interpretable, and transparently artificial.

Future studies should examine how robot speech variability interacts with multimodal cues (e.g., gaze, gesture, timing) to shape trust and developmental outcomes, including linguistic and cultural diversity (e.g., Andrist et al., 2015), while minimizing bias and stereotypes, and utilizing cross-linguistic and longitudinal designs. Research should also extend beyond dyadic interactions to polyadic settings with multiple children, caregivers, or educators, investigating bidirectional speech adaptation in these socially shared contexts. Importantly, more research is needed to understand the conditions under which children recognize synthetic speech as artificial and how this metacognitive awareness develops with age.

From a technical feasibility perspective, advancing robot speech varieties requires generative models trained on child-directed, multimodal speech and improved child-speech recognition systems that can handle dialectal and developmentally variable input. While neural TTS synthesis steadily improve, fully autonomous bidirectional adaptation between children and robots remains technically challenging. Moreover, adaptive control of prosody, clarity, and timing could improve accessibility for children with hearing, speech, or neurodevelopmental differences, allowing robot varieties to serve a broader range of learners and make CRI more inclusive.

Rather than aiming to make robots sound perfectly human, the future of CRI should treat developmental appropriateness as the primary criterion for vocal design. This shift reframes variability as a design resource to be deployed selectively, transparently, and in alignment with children’s learning needs, rather than as a by-product of human-likeness. Meeting these challenges will require collaboration across diverse disciplines and cultural contexts.

## Data Availability

The original contributions presented in the study are included in the article/supplementary material, further inquiries can be directed to the corresponding author.
